# Comparative analyses of the metabolite and ion concentrations in nectar, nectaries, and leaves of 36 bromeliads with different photosynthesis and pollinator types

**DOI:** 10.3389/fpls.2022.987145

**Published:** 2022-08-26

**Authors:** Thomas Göttlinger, Gertrud Lohaus

**Affiliations:** Molecular Plant Science and Plant Biochemistry, University of Wuppertal, Wuppertal, Germany

**Keywords:** Bromeliaceae, floral nectar, nectaries, nectar composition, sugar transport, amino acids, inorganic ions

## Abstract

Floral nectar contains mainly sugars as well as smaller amounts of amino acids and further compounds. The nectar composition varies between different plant species and it is related to the pollination type of the plant. In addition to this, other factors can influence the composition. Nectar is produced in and secreted from nectaries. A few models exist to explain the origin of nectar for dicotyl plant species, a complete elucidation of the processes, however, has not yet been achieved. This is particularly true for monocots or plant species with CAM photosynthesis. To get closer to such an elucidation, nectar, nectaries, and leaves of 36 bromeliad species were analyzed for sugars, starch, amino acids, and inorganic ions. The species studied include different photosynthesis types (CAM/C3), different pollination types (trochilophilous/chiropterophilous), or different live forms. The main sugars in nectar and nectaries were glucose, fructose, and sucrose, the total sugar concentration was about twofold higher in nectar than in nectaries, which suggests that sugars are actively transported from the nectaries into the nectar. The composition of amino acids in nectar is already determined in the nectaries, but the concentration is much lower in nectar than in nectaries, which suggests selective retention of amino acids during nectar secretion. The same applies to inorganic ions. Statistical analyses showed that the photosynthesis type and the pollination type can explain more data variation in nectar than in nectaries and leaves. Furthermore, the pollinator type has a stronger influence on the nectar or nectary composition than the photosynthesis type. Trochilophilous C3 plants showed significant correlations between the nitrate concentration in leaves and the amino acid concentration in nectaries and nectar. It can be assumed that the more nitrate is taken up, the more amino acids are synthesized in leaves and transported to the nectaries and nectar. However, chiropterophilous C3 plants show no such correlation, which means that the secretion of amino acids into the nectar is regulated by further factors. The results help understand the physiological properties that influence nectaries and nectar as well as the manner of metabolite and ion secretion from nectaries to nectar.

## Introduction

Floral nectar is a biochemical complex solution and nectar composition varies between plant species. Nectar is produced in nectaries and secreted from this tissue. Sugars, mainly the hexoses glucose and fructose, as well as the disaccharide sucrose, are dominant. Besides, nectar contains numerous other compounds, including amino acids, inorganic ions, organic acids, and further secondary compounds, albeit in much lower concentrations than sugars (Baker and Baker, 1973; Adler, 2000; Heil, 2011; Lohaus and Schwerdtfeger, 2014; Nicolson, 2022). The main function of nectar is to attract the relevant pollinators for the particular plant species and to have an impact on plant-pollinator interactions, but also to protect from potential herbivores (Proctor et al., 1996; Kessler et al., 2012; Nicolson, 2022).

Nectar-sugars are the main source of energy for pollinators and the sucrose-to-hexoses ratios have often been related to the pollination type of the plant species (Baker and Baker, 1983; Krömer et al., 2008; Tiedge and Lohaus, 2017; Göttlinger et al., 2019). In some studies, the concentrations of amino acids or inorganic ions in nectar were also related to the respective pollinators, which was shown for several species of the genus *Nicotiana* (Tiedge and Lohaus, 2017) or numerous species of Bromeliaceae (Göttlinger et al., 2019). Amino acids in nectar are an important source of nitrogen for some flower visitors like many adult insects; hummingbirds, however, are primarily insect-catchers, and bats also use insects and pollen as an additional nitrogen source (Baker, 1977; Herrera et al., 2001). Therefore, these vertebrates are not dependent on nectar as sole nitrogen source (Baker, 1977). Inorganic ions in nectar are believed to affect the electrolytic balance of the visitors (Hiebert and Calder, 1983). However, until now interactive effects of different nectar compounds on pollinator responses have rarely been considered (Nicolson, 2022).

The plant family of Bromeliaceae is one of the largest in the tropical and subtropical America (Benzing, 2000). Bromeliads are adapted to a wide range of different habitats with various environmental conditions and therefore they are very diverse in physiological, ecological or morphological aspects. More than half of the species are epiphytes and the others show terrestrial live-forms (Zotz, 2013). The type of photosynthesis is distributed comparingly: almost half of the species use the Crassulacean Acid Metabolism (CAM) photosynthesis pathway and the other half the C3 photosynthesis pathway (Crayn et al., 2015). However, the relative abundance of the photosynthesis pathway is very different within the genera; e.g., almost all analyzed species of the genera *Aechmea*, *Billbergia*, or *Quesnelia* show CAM photosynthesis, whereas the species of the genera *Alcantarea*, *Pitcairnia*, or *Werauhia* show only C3 photosynthesis (Crayn et al., 2015). Most bromeliads are pollinated by vertebrates, either hummingbirds or bats (Benzing, 2000; Krömer et al., 2006; Aguilar-Rodríguez et al., 2019). Bat-pollinated (chiropterophilous) species are usually species with C3 photosynthesis, whereas hummingbird-pollinated (trochilophilous) bromeliads show either C3 or CAM photosynthesis (Pierce et al., 2002).

In bromeliads, floral nectar is produced by septal nectaries, which represents an anomaly in the order Poales (Benzing, 2000). The nectaries are located in the basal part of the ovary and they are formed by an incomplete fusion of the carpels (Benzing, 2000). In the secretory phase, the nectaries comprise two regions, an epithelium and the nectary parenchyma, which together form nectar-secreting channels (Bernadello et al., 1991; Stahl et al., 2012). The tissue has a labyrinthine surface with vascular bundles, which facilitates increased nectar production for special pollinators such as bats (Sajo et al., 2004).

In general, several metabolic steps are involved in the production of sugars in the nectaries, and there are different models to describe the production and secretion of the floral nectar. According to the model of Vassilyev (2010), nectar moves in the apoplast (around parenchymal cells) to the nectary surface by a pressure-driven mass flow. Furthermore, pre-nectar sugars diffuse from the phloem to secretory cells in the symplast *via* plasmodesmata, where sugars are actively transported across the plasma membrane into the apoplast (Vassilyev, 2010). Another model of the eccrine-based nectar secretion includes the import of sucrose delivered from the phloem (Lin et al., 2014), accumulation of starch in the nectary parenchyma cells (Pacini et al., 2003), starch degradation at anthesis (Peng et al., 2004; Ren et al., 2007), sucrose synthesis (Lin et al., 2014), as well as in the export of sucrose from the nectaries by the uniporter Sugar Will Eventually be Exported Transporter (SWEET9; Lin et al., 2014). This transporter could also contribute to glucose efflux (Lin et al., 2014). Furthermore, the proportion of hexoses in nectar is related to the activity of sucrose-cleavage enzymes (e.g., cell wall invertases), other metabolic processes during nectar production, or transport processes during nectar secretion (Ruhlmann et al., 2010; Tiedge and Lohaus, 2018). In addition to this eccrine-based nectar secretion, granulocrine-based nectar secretion was proposed for some bromeliads as well, based on the ultrastructure of the secretory epithelia (Stahl et al., 2012; Mosti et al., 2013). According to this model, metabolites are transported symplastically to the cells of the nectary surface. Here, they are stored in vesicles and secreted into the nectar by fusion with the plasma membrane (Fahn, 1979). Although these models already allow for a good insight into the mechanisms involved, a complete elucidation of the processes involved in nectar production and secretion has not yet been achieved (Roy et al., 2017). Moreover, in addition to nectar production and secretion, nectar is also reabsorbed by nectaries which makes the whole process even more complex (Nepi and Stpiczyñska, 2008).

Most analyses of the sugar production and secretion were done on *Arabidopsis*, *Brassica* or *Nicotiana* ssp., whereas the knowledge about other plant species, such as monocots or species with CAM photosynthesis, remains incomplete. Even less is known about the origin, production and secretion of non-carbohydrate compounds in nectar, such as amino acids or inorganic ions, or about the corresponding concentrations of such compounds in nectaries (Solhaug et al., 2021). Moreover, studies focused on the nectaries only, which means that knowledge about the influence of the pollinator type or the photosynthesis type on this nectar producing tissue is scarce (Tiedge and Lohaus, 2018).

To get further insight into the origin of nectar compounds and the mechanisms that determin nectar composition, the main metabolites and ions in nectar and nectaries of 36 bromeliad species were compared. These species were selected because they differ in the type of photosynthesis (CAM or C3) as well as in the plant’s pollinator type (trochilophilous or chiropterophilous). Furthermore, the metabolites and ions were analyzed in the leaves of the bromeliads in pursuit of the question whether the nectar and/or nectary composition correlates with the metabolite and ion contents in the whole plant.

We hypothesize that the nectar composition is already prescribed by the metabolite and ion composition in the nectaries. Furthermore, both the photosynthesis type and pollination type of the plant species influence the composition in nectar as well as in nectaries. Finally, increased metabolite contents in the whole plant may also result in increased contents in nectaries as well as in nectar.

## Materials and methods

### Plant material

The plant material and nectar samples were obtained from bromeliads grown in tropical glasshouses in the Botanical Garden and Botanical Museum Berlin (Germany), the Botanical Garden of the University of Göttingen (Germany), the Botanical Garden of the University of Heidelberg (Germany) and at the University of Wuppertal (Germany).

For the experiments, 36 bromeliad species of three subfamilies (Bromelioideae, Pitcairnioideae, Tillandsioideae) and 12 genera (*Aechmea*, *Alcantarea*, *Billbergia*, *Guzmania*, *Lutheria*, *Pitcairnia*, *Portea*, *Pseudalcantarea*, *Quesnelia*, *Tillandsia*, *Vriesea*, *Werauhia*) were used. The species differ in the type of photosynthesis (14 species with CAM and 22 species with C3 photosynthesis) and the pollination type (25 trochilophilous and 11 chiropterophilous species; Supplementary Table 1).

### Collection of nectar, nectaries, and leaves

For each of the 36 species, at least three plants were used for the experiments. Nectar as well as nectaries and leaves were harvested from day-pollinated (trochilophilous) and night-pollinated (chiropterophilous) bromeliads shortly after anthesis. Nectar samples (5–10 μl) were collected from single flowers with the help of a micropipette (Göttlinger et al., 2019). As periodic removal is known to affect nectar volume and concentration (Galetto and Bernardello, 1992), nectar was only sampled once per flower. At least three independent nectar samples from three plants were collected for each bromeliad species. According to Sajo et al. (2004), the septal nectary was dissected from the ovaries of each flower using a binocular microscope. External sugars were removed from the tissue by rinsing the nectaries with ultrapure water. Three independent nectary samples were collected from three plants of the same species. Since the analysis of nectary tissues requires about 25 mg tissue per sample, nectary tissue from 10 to 20 flowers per plant had to be pooled, depending on the size of the nectaries in the various species. One leaf sample per plant from three different plants of the same species were taken from the central area of a medium-aged leaf using a razor blade. Consequently, a minimum of three samples per tissue (leaf, nectary, nectar) of three different plants were collected from each bromeliad species at the same time. The samples were frozen immediately in liquid nitrogen and stored at –80°C until further analysis.

### Extraction of soluble metabolites from nectary and leaf tissue

Soluble metabolites or inorganic ions were extracted using finely milled powder of 25 mg nectary tissue or 200 mg leaf tissue by chloroform-methanol-water extraction (Göttlinger and Lohaus, 2020).

### Analyses of sugars in nectar, nectary, and leaf tissue

The analyses of sugars were performed *via* HPLC (Thermo Fisher Scientific Dionex ICS-5000 HPIC System) according to Lohaus and Schwerdtfeger (2014). The sugars were eluted isocratically using an anion exchange column (Dionex™ CarboPac™ PA10 4 × 250 mm) and detected with a pulse amperometric detector. The sugar concentrations in the samples were determined using calibration curves for the different sugars. An integration program (Chromeleon 7.2) was used to evaluate the chromatograms using the calibration curves. The sugar concentrations in nectar are given in millimolar (mM), and in the extracts of nectaries and leaves in μmol⋅g^–1^ fresh weight (FW). Using the water content of leaf cells (86%) and nectary cells (75%; Tiedge and Lohaus, 2018), metabolite and ion concentrations in these tissues can be expressed in millimolar (mM) as well.

### Analyses of free amino acids in nectar, nectary, and leaf tissue

The concentrations of free amino acids in nectar, nectaries, and leaves were determined *via* HPLC (Thermo Fisher Scientific UltiMate 3000) according to Göttlinger et al. (2019). The concentration of 19 amino acids (alanine, arginine, aspartate, asparagine, glutamate, glutamine, glycine, histidine, isoleucine, leucine, lysine, methionine, phenylalanine, proline, serine, threonine, tryptophan, tyrosine, valine) were detected with a fluorescence detector after separation on a reversed-phase column (Merck LiChroCART^§^ 125-4 using Superspher^§^ 100 RP-18 endcapped). The concentrations in nectar, nectary tissue, and leaf tissue were determined using calibration curves for the different amino acids (Chromeleon 7.2). The amino acid concentrations in nectar are given in millimolar (mM) and in the nectary and leaf tissues in μmol⋅g^–1^ fresh weight (FW). Using the water content of leaf cells (86%) and nectary cells (75%; Tiedge and Lohaus, 2018), metabolite and ion concentrations in these tissues can be expressed in millimolar (mM) as well.

### Analyses of inorganic ions in nectar, nectary, and leaf tissue

The inorganic ions include anions (chloride, nitrate, phosphate, sulfate) and cations (potassium, sodium, magnesium, calcium), their concentrations in nectar, nectaries, and leaves were determined separately *via* HPLC according to Lohaus et al. (2001). The ions were eluted isocratically either by an anion exchange (Dionex™ IonPac™ AS11 4 × 250 mm) or cation exchange column (Dionex™ CS 12A, 4 × 250 mm) and detected by their electronic conductivity. External calibration standards for each ion were measured in parallel. The concentrations of inorganic anions and cations in nectars are given in millimolar (mM) and in the tissues in μmol⋅g^–1^ fresh weight (FW). Using the water content of leaf cells (86%) and nectary cells (75%; Tiedge and Lohaus, 2018), metabolite and ion concentrations in these tissues can be expressed in millimolar (mM) as well.

### Analyses of starch in nectary tissue and leaf tissue

In nectaries and leaf tissues the content of starch was analyzed according to Riens et al. (1994). After treatment with potassium hydroxide, α-amylase, and amyloglucosidase the released glucose was measured enzymatically.

### Statistical analysis

All statistical analyses were performed using R (version 4.1.2).^1^ The metabolite and ion compositions of nectar, nectary tissue, and leaf tissues in 36 bromeliad species with different photosynthesis types (C3, CAM) as well as pollination types (trochilophilous, chiropterophilous) were compared using an ANOVA followed by a *post-hoc* test (Tukey; *p*-value < 0.05). Further, to determine whether there is a significant difference between two groups of samples, *t*-tests were applied.

The influences of the photosynthesis and pollinator type on the composition were examined using a Principal Component Analysis (PCA) according to Göttlinger et al. (2019). For the determination of the influence of the photosynthesis type, five CAM- and five C3-bromeliad were included in the analysis. To decrease the influence of the genera on the data, plant species from different genera were chosen. In the PCA for pollinator type, seven bat- and seven hummingbird-pollinated species were used. All the chosen plant species show C3 metabolism to eliminate the influence of different photosynthesis types. The seven different species originate from genera including both trochilophilous and chiropterophilous species. For this purpose, *Alcantarea*, *Guzmania*, *Pitcairnia*, *Tillandsia*, and *Vriesea* were examined. The selection of data resulted in a balanced design, the heterogeneity of which is very robust in Permutational Multivariate Analysis of Variance (PERMANOVA) (Anderson, 2014), which was performed according to Göttlinger et al. (2019). To identify the relative importance of the variables on the nectar composition, the pollinator type or photosynthesis type and, respectively associated taxonomic groups were used. In addition, Permutational Analysis of Multivariate Dispersions (PERMDISP) was performed to test the extent to which significance in PERMANOVA is caused by location and dispersion effects (Anderson, 2006). The analysis was performed by using the “vegan” package in R based on Euclidean distance measures with the *betadisper* routine (Oksanen et al., 2007).

The normal distribution of the samples was confirmed by quantile-quantile plots. According to existing normal distribution, a Pearson’s rank correlation was performed in order to test whether a relationship exists between the different components (sugars, starch, amino acids, inorganic ions) in leaves, nectaries, and nectar. Simultaneously, the Pearson correlation coefficient r was determined. To calculate the correlation matrix, the “cor” function in R was used. The correlation matrices were visualized as heatmaps with a considered significance for *p* ≤ 0.001 using the “corrplot” package (Wei and Simko, 2021). To distinguish the differences between the various groups of bromeliads, the correlation matrix was created with all 36 bromeliad species as well as only with those species with different photosynthesis or pollinator types.

## Results

### Comparison of leaf, nectary, and nectar composition

The concentrations of sugars, amino acids, and inorganic ions in nectar, nectaries, and leaves of 36 bromeliad species (Supplementary Table 1) were determined. In addition, the metabolite and ion concentrations of the plants with different photosynthesis type (C3, CAM) or pollinator type (Tro = trochilophilous, Chi = chiropterophilous) were compared with each other. As all sampled CAM species have trochilophilous pollinators, only such species with trochilophilous pollinators and C3 metabolism were included in the analyses of the influence of the photosynthesis type to minimize the simultaneous influence of the pollinator type. For the analysis of the influence of the pollinator type, only C3 plants were included which have either trochilophilous or chiropterophilous pollinators to minimize the influence of the photosynthesis type.

### Sugars in leaves, nectaries, and nectar

**FIGURE 1:**
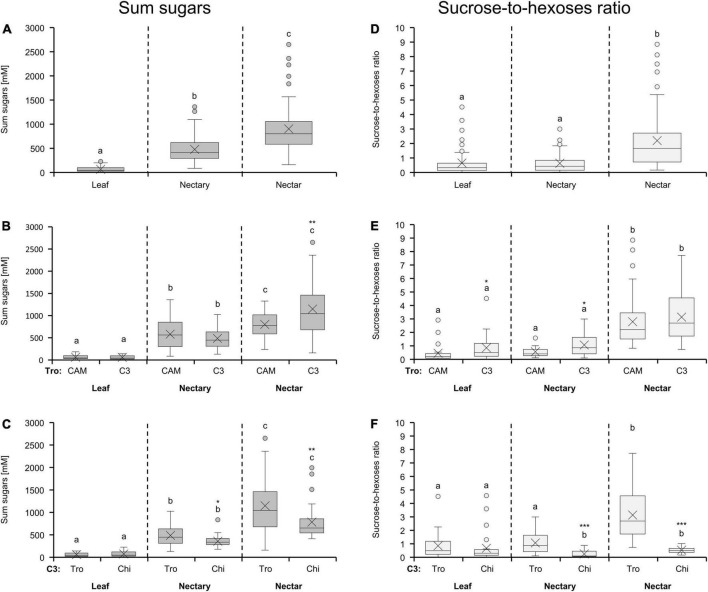
Sum of sugars (glucose, fructose and sucrose) and sucrose-to-hexoses ratios in leaf, nectary and nectar **(A,D)**. The shown data includes all 36 bromeliad species (*n* = 3). Additionally, the data were separated by photosynthesis type **(B,E)** and pollinator type **(C,F)** in the boxplot diagrams. The separation according to photosynthesis type includes 14 species with CAM photosynthesis and 22 species with C3 photosynthesis. All CAM species have trochilophilous pollinators whereas the C3 species have either trochilophilous (tro) or chiropterophilous (chi) pollinators. Therefore, the C3 species were separated according to the pollinator type in 11 trochilophilous (tro) and 11 chiropterophilous (chi) species. Different letters represent significant differences in sum of sugars or sucrose-to-hexoses ratios, respectively, between leaf, nectary and nectar (Tukey’s HSD; *p* < 0.05). The asterisks show different levels of significance between the photosynthesis or pollinator types, respectively (*p < 0.05, **p < 0.01, ***p < 0.001).

Leaves, nectaries, and nectar of all 36 bromeliads contain glucose, fructose, and sucrose. The concentration of the sum of sugars was highest in nectar, followed by the nectaries, and lowest in leaves (Figure 1A). The mean sugar concentration was about sevenfold higher in nectary (477 mM) than in leaf tissue (64 mM) and about twofold higher in nectar (900 mM) than in nectaries (477 mM). This rank of sugar concentrations in the different tissues/solutions was also found for the bromeliads with different photosynthesis types (Figure 1B) as well as different pollinator types (Figure 1C). The sum of sugars in nectar was significantly higher in C3 than in CAM species (*p* < 0.01), whereas the sugar concentrations in leaves as well as in nectaries of both photosynthesis types were similar (Figure 1B). For both pollinator types, the sugar concentrations in nectar was generally higher than in nectaries, however, the comparably lower sugar concentration in nectar of chiropterophilous pollinated plants was already apparent in nectary tissue (Figure 1C).

Considering all 36 bromeliads, the sucrose-to-hexoses ratio in leaves and nectaries were comparable, whereas in nectar, the ratio was increased. This means that nectar contained more sucrose than hexoses (Figure 1D). The sucrose-to-hexoses ratio was slightly higher in leaves, nectaries, and nectar of C3 species compared to CAM species (Figure 1E). When comparing plants with different pollination types, the sucrose-to-hexoses ratios in nectar as well as in nectaries of chiropterophilous species was significantly lower than in trochilophilous species (*p* < 0.001; Figure 1F). This difference can also be found in leaves, albeit not on a significant level (Figure 1F).

### Amino acids

**FIGURE 2:**
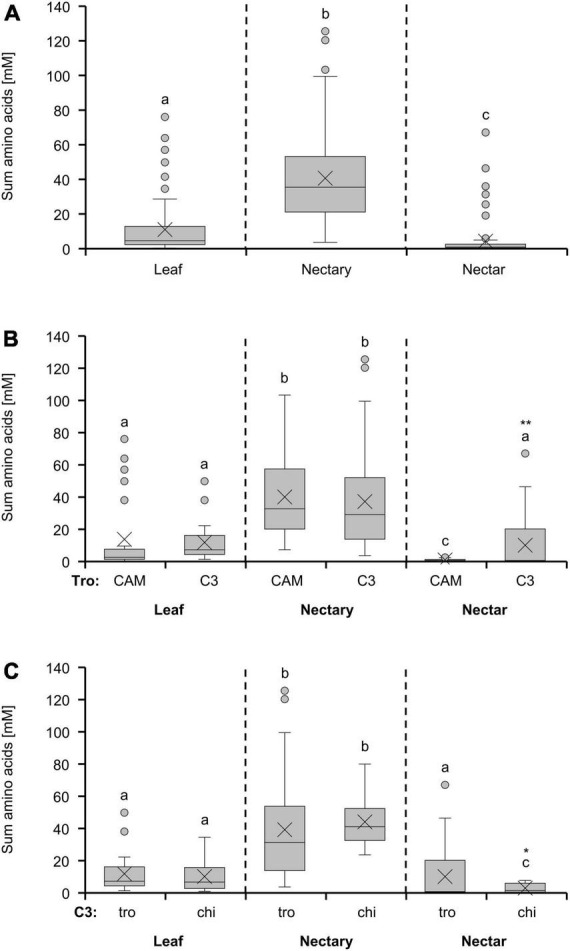
Sum of 19 amino acids in leaf, nectary and nectar additionally depicted as boxplot diagram. The first diagram **(A)** shows data including all 36 bromeliad species (*n* = 3). Additionally, the data were separated by photosynthesis type **(B)** and pollinator type **(C)**. The separation according to photosynthesis type includes 14 CAM and 22 C3 species. All CAM species have trochilophilous pollinators whereas the C3 species have either trochilophilous (tro) or chiropterophilous (chi) pollinators. Therefore, the C3 species were separated according to the pollinator type in 11 trochilophilous (tro) and 11 chiropterophilous (chi) species. Different letters represent significant differences in sum of amino acids between leaf, nectary and nectar (Tukey’s HSD; *p* < 0.05). The asterisks show different levels of significance between the photosynthesis and pollinator types, respectively (*p < 0.05, **p < 0.01).

The highest amino acid concentration was found in nectaries, followed by leaves, and the lowest concentration was found in nectar (Figure 2A). When comparing CAM and C3 species, the amino acid concentration in leaves and nectaries of both photosynthesis types was similar (Figure 2B). A significant difference could only be detected in nectar of the two photosynthesis types, with the concentration in nectar of C3 species being significantly higher than in nectar of CAM species (*p* < 0.01; Figure 2B). The comparison of plants in the light of the two pollination types trochilophilous or chiropterophilous reveals a significantly lower concentration in nectar of chiropterophilous species (*p* < 0.05; Figure 2C), whereas in leaves or nectaries no differences were found between the two pollination types.

Leaves, nectaries, and nectar of the different groups of bromeliads also showed different ratios sum-of-sugars-to-sum-of-amino-acids. The ratios were roughly between 10 and 50 in leaves, 9 and 25 in nectaries, and 1,042 and 2,199 in nectar (Table 1). This means that nectar contains much more sugar in relation to amino acids than to nectaries and leaves. The ratios were higher in nectaries and nectar of C3 plants (trochilophilous pollination) compared to the other bromeliad groups, which means that nectaries and nectar of C3 plants contain the most sugar in relation to amino acids.

**TABLE 1:** The ratio of sum-of-sugars-to-sum-of-amino-acids in leaf, nectary and nectar divided according to photosynthesis and pollinator type.

Σ Sugars/Σ Amino acids		Leaf	Nectary	Nectar
All	(*n* = 36)	28.8 ± 53.0	17.9 ± 17.8	1791.9 ± 3259.6
CAM (tro)	(*n* = 14)	49.8 ± 76.7	19.5 ± 17.3	2061.8 ± 3970.8
C3-tro	(*n* = 11)	10.0 ± 9.1	24.9 ± 22.8	2198.8 ± 3541.1
C3-chi	(*n* = 11)	20.9 ± 27.4	8.7 ± 3.4	1041.6 ± 1393.1

Data from Figures 1, 2.

**FIGURE 3:**
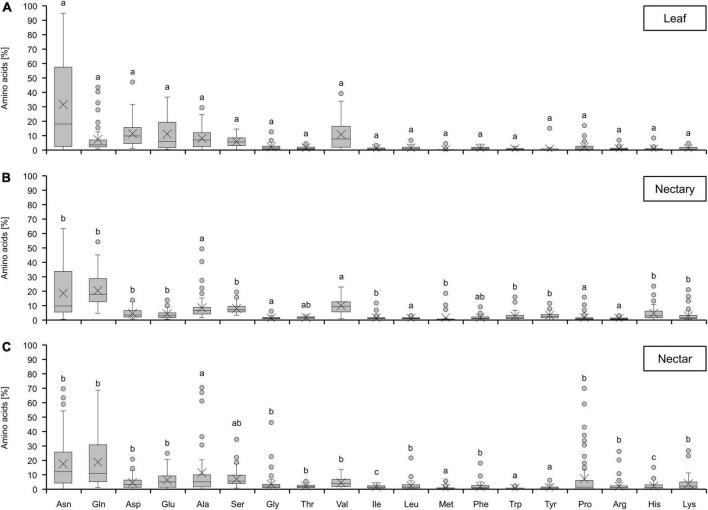
Percentage of different amino acids in leaf **(A)**, nectary **(B)** and nectar **(C)**. The shown data includes all 36 bromeliad species (*n* = 3). Different letters represent significant differences in individual amino acids between leaf, nectary and nectar (Tukey’s HSD; *p* < 0.05).

In leaves, nectaries, and nectar of all bromeliad species, 19 amino acids were found and the similarity of the amino acid composition in the different tissues is obvious (Figure 3). In each case, the main amino acids were asparagine, glutamine, aspartate, glutamate, alanine, serine, and valine. The proportions of other amino acids, such as methionine or tryptophan, were very low. When comparing nectaries and nectar, no significant differences could be detected for most of the amino acids. However, some amino acids were more abundant in nectar than in nectaries, e.g., proline and glycine. Similar results were also found for species with either CAM or C3 photosynthesis (Supplementary Figure 1) as well as different pollination types (Supplementary Figure 2).

### Inorganic ions

**FIGURE 4:**
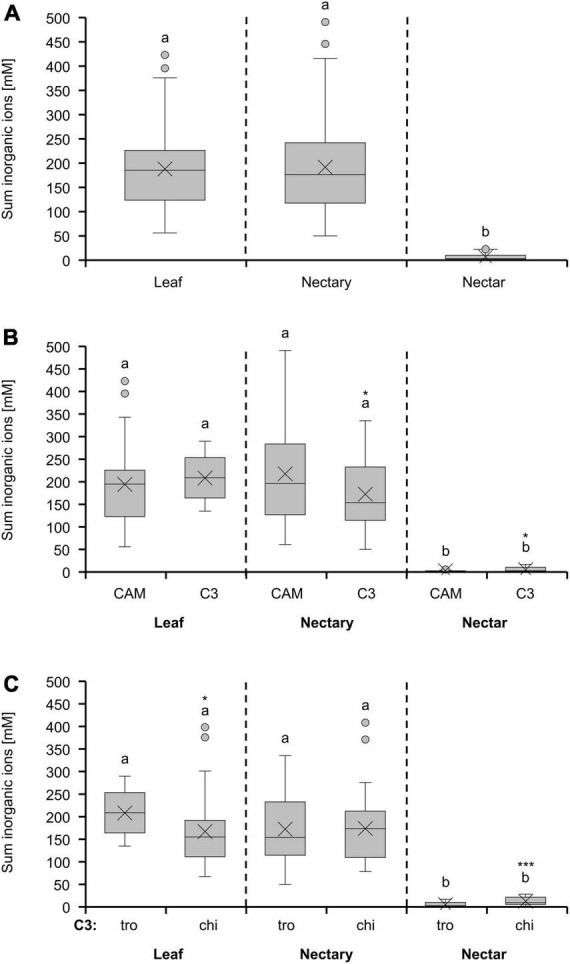
Sum of 8 inorganic ions (anions: Cl^–^, PO_4_^3–^, SO_4_^2–^, NO_3_^–^; cations: K^+^, Na^+^, Mg^2+^, Ca^2+^) in leaf, nectary and nectar. Boxplot diagram **(A)** shows data including all 36 bromeliad species (*n* = 3). Additionally, the data were separated by photosynthesis type **(B)** and pollinator type **(C)**. The separation according to photosynthesis type includes 14 CAM and 22 C3 species. All CAM species have trochilophilous pollinators whereas the C3 species have either trochilophilous (tro) or chiropterophilous (chi) pollinators. Therefore, the C3 species were separated according to the pollinator type in 11 trochilophilous (tro) and 11 chiropterophilous (chi) species. Different letters represent significant differences in sum of amino acids between leaf, nectary and nectar (Tukey’s HSD; *p* < 0.05). The asterisks show different levels of significance between the photosynthesis and pollinator types, respectively (*p < 0.05, ***p < 0.001).

The concentrations of inorganic ions in leaves and nectaries of the bromeliad species were similar, whereas the concentration in nectar was about 30-fold lower (Figure 4A). A similar pattern was observed for the different photosynthesis types (Figure 4B) or pollination types (Figure 4C). Furthermore, C3 plants with trochilophilous pollination and especially C3 plants with chiropterophilous pollination showed significantly higher levels of inorganic ions in nectar than CAM plants (*p* < 0.05; Figure 4B and textitp < 0.001; Figure 4C).

Leaves, nectaries, and nectar of the different groups of bromeliads also showed different ratios sum-of-sugars-to-sum-of-inorganic-ions. The ratios were between 0.3 and 0.5 in leaves, 2.5 and 3.3 in nectaries, and 110 and 732 in nectar (Table 2). This means that nectaries and especially nectar contains more sugar in relation to inorganic ions, whereas leaves contain less sugar than inorganic ions. The ratio was higher in nectaries and nectar of C3 plants with trochilophilous pollination compared to the other bromeliad groups, which means that nectar of these plants contains more sugar in relation to inorganic ions. Particularly nectaries and nectar of C3 plants with chiropterophilous pollination contains relatively more inorganic ions.

**TABLE 2:** The ratio of sum-of-sugars-to-sum-of-inorganic-ions in leaf, nectary and nectar divided according to photosynthesis and pollinator type.

Σ Sugars/Σ Ions		Leaf	Nectary	Nectar
All	(*n* = 36)	0.4 ± 0.4	2.9 ± 1.7	478.2 ± 663.8
CAM (tro)	(*n* = 14)	0.4 ± 0.4	2.9 ± 1.7	568.2 ± 452.2
C3-tro	(*n* = 11)	0.3 ± 0.2	3.3 ± 2.0	731.5 ± 992.0
C3-chi	(*n* = 11)	0.5 ± 0.5	2.5 ± 1.4	110.2 ± 104.3

Data from Figures 1, 3.

**FIGURE 5:**
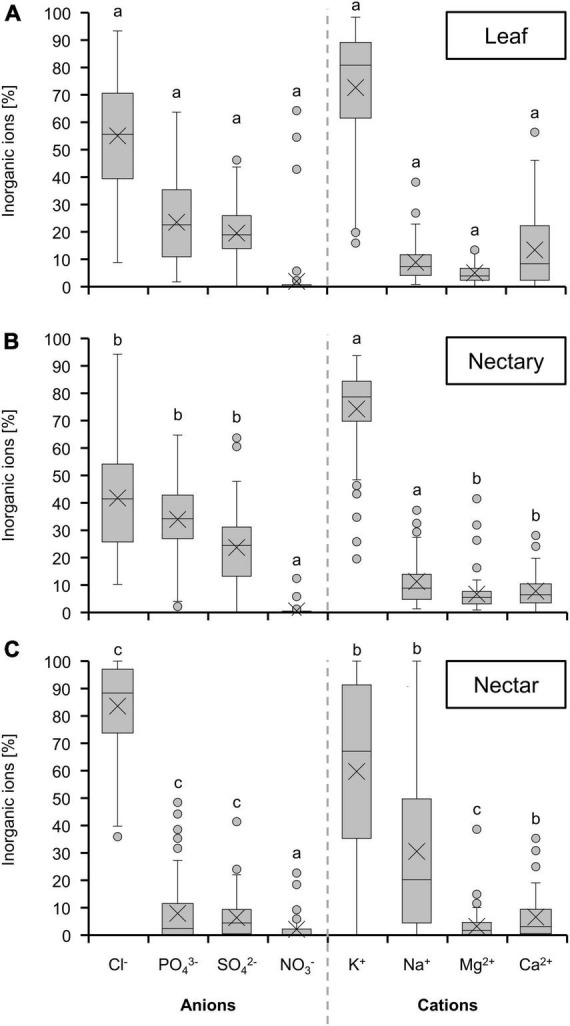
Percentage of different inorganic ions in leaf **(A)** nectary **(B)** and nectar **(C)**. The shown data includes all 36 bromeliad species (*n* = 3). Different letters represent significant differences in individual amino acids between leaf, nectary and nectar (Tukey’s HSD; *p* < 0.05).

The composition of inorganic ions was relatively similar in leaves and nectaries (Figures 5A,B). Chloride was the most abundant anion, followed by phosphate, sulfate, and nitrate as the lowest concentrated anion. Among the cations, potassium was dominant, with the proportions of sodium, magnesium and calcium being much lower. In nectar, the proportion of chloride was higher compared to nectaries and leaves (Figure 5). The proportions of potassium and sodium were very variable, and the proportion of sodium was generally higher in nectar than in nectaries or leaves. Similar results could be obtained when comparing the species in the light of photosynthesis (Supplementary Figure 3) or pollination types (Supplementary Figure 4).

### Influence of photosynthesis type and pollination type on leaf, nectary, and nectar composition of sugars, amino acids, and inorganic ions

In order to investigate whether the differences in metabolite or ion concentrations could be explained by the photosynthesis type of the bromeliad species, PCA were performed. All species which are included in the analyses belong to the trochilophilous pollination type to reduce the influence of other factors besides the photosynthesis type. The principal components of nectar explained 45%, of nectaries 58%, and of leaves 48% of the total variance of the data (Supplementary Figures 5A,C,E). The scatterplot of this PCA of leaves and nectar show a visual separation of the photosynthesis types (Supplementary Figures 5B,F).

The leaf, nectary, and nectar compositions with regard to the respective components of 14 species with either trochilophilous or chiropterophilous pollination from five different genera were selected to analyze the influence of the pollination type by PCA (Supplementary Figure 6). All selected species belong to the C3 photosynthesis type to reduce the influence of other factors besides the pollination type. The scatterplot of nectaries and nectar (Supplementary Figures 6B,D) show that the species can be visually separated on the basis of the pollinator type. In the case of leaves, 45%, of nectaries 37%, and of nectar 43% of the variance can be explained by the principal components.

To evaluate the graphical results, a PERMANOVA followed by PERMDISP was performed using genus and photosynthesis type or genus and pollination type as categorical variables (Table 3). In the case of nectar, the photosynthesis type as categorical variable of the PERMANOVA can explain 25% of the data variation on a significant level (*p* < 0.001), and the pollinator type 35% of the data variation also on a significant level (*p* < 0.001). The category genus explains 51% (*p* < 0.001) for the variable photosynthesis type and 16% (*p* < 0.05) for the variable pollinator type. However, the significant *p*-value (*p* < 0.001) for the category genus in PERMDISP indicates that significant *p*-values in PERMANOVA cannot be considered (Table 3). For nectaries, the influence explaining the data variation decreases to 12% (*p* < 0.05; photosynthesis) and 22% (*p* < 0.001; pollinator). Considering the leaf samples, the lowest explanation of data variation can be found by photosynthesis and pollinators, each at 9%. However, PERMDISP (*p* < 0.05) indicates mostly that the influence of genus is caused by location and dispersion effects. The percentage explained by the categories photosynthesis and pollinator decreases accordingly from nectar to the nectaries and further to the leaves, with the higher influence of the pollinator type compared to the photosynthesis type in nectar and nectaries (Table 3).

**TABLE 3:** Results of the PERMANOVA and PERMDISP: Degrees of freedom (df), pseudo-F (F), *R*^2^, and *p*-values.

	Degrees of freedom (df)	Pseudo-F (F)	*R* ^2^	PERMANOVA *P*-value	PERMDISP *P*-value
**Nectar**					
Photosynthetic type	1	24.11	0.25	0.001***	0.173
Genus	6	8.14	0.51	0.001***	0.046*
Residuals	22		0.23		
Total	29		1.00		
Pollinator	1	79.06	0.35	0.001***	0.060
Genus	4	15.83	0.28	0.001***	0.013*
Residuals	24		0.37		
Total	29		1.00		
**Nectary**					
Photosynthetic type	1	3.82	0.12	0.02*	0.094
Genus	6	1.13	0.21	0.34	0.001***
Residuals	22		0.67		
Total	29		1.00		
Pollinator	1	25.79	0.22	0.001***	0.070
Genus	4	10.66	0.37	0.001***	0.047*
Residuals	24		0.41		
Total	29		1.00		
**Leaf:**					
Photosynthetic type	1	6.28	0.09	0.001***	0.676
Genus	6	6.94	0.60	0.001***	0.001***
Residuals	22		0.31		
Total	29		1.00		
Pollinator	1	16.16	0.09	0.001***	0.159
Genus	4	22.04	0.50	0.001***	0.808
Residuals	24		0.41		
Total	29		1.00		

R^2^ describes the influence of the photosynthetic type, the genus and the pollinator on the nectar, nectary and leaf composition.

For the statistical analysis of the photosynthesis type, only trochilophilous species from different genera were selected. CAM: Aechmea gamosepala, Billbergia vittata, Quesnelia quesneliana, Tillandsia flabellata, Tillandsia funckiana; C3: Alcantarea regina, Guzmania melinonis, Pitcairnia maidifolia, Pitcairnia olivia-estevae, Vriesea guttata. For the statistical analysis of the pollinator, only C3-species from different genera were selected based on the pollinator. Chi: Alcantarea imperialis, Guzmania calothyrsus, Pitcairnia wendlandii, Tillandsia rauhii, Vriesea unilateralis; Tro: Alcantarea regina, Guzmania melinonis, Pitcairnia maidifolia, Tillandsia malzinei, Vriesea guttata.

The asterisks show different level of significance (*p < 0.05, ***p < 0.001).

### Correlation of leaf, nectary, and nectar components

**FIGURE 6:**
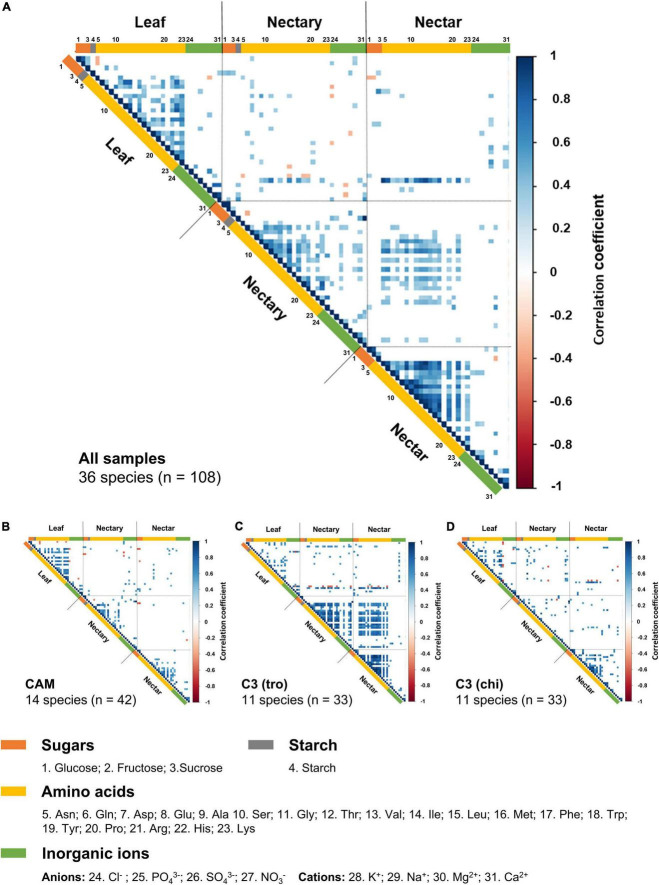
Correlation heatmap for sugars, starch, amino acids, inorganic ions in leaf, nectary and nectar. The scale is colored for the value of the Pearson’s correlation coefficient. Blue represents a positive correlation while red represents negative correlation. The values of 1 and –1 marks each a perfect correlation. The correlation heatmap is divided according to leaf, nectaries and nectar. The numbers at the listed components indicate the order of the components in the correlation heatmap. Blank white boxes show non-significant correlations (*n* = 3; *p* < 0.001). The first correlation heatmap **(A)** represents the complete data (36 species, *n* = 108). The smaller heatmaps show data from species with CAM-photosynthesis **(B,** 11 species, *n* = 33) or with C3-photosynthesis and trochilophilous pollination **(C,** 11 species, *n* = 33) or with C3-photosynthesis and chiropterophilous pollination **(D**, 11 species, *n* = 33).

For further analyses of the relations between the concentrations of the different sugars, amino acids, and inorganic ions in leaves, nectaries, and nectar, correlation matrices was created with the data of all 36 species (Figure 6A), data of species according to the photosynthesis types (Figures 6B,C) and data of species according to the pollination types (Figures 6C,D).

Considering all 36 species (Figure 6A), a positive correlation was found between the starch content in leaves and the sugar concentrations in nectaries (*r* = 0.39–0.45; *p* < 0.001), as well as the sucrose concentration in nectar (*r* = 0.33; *p* < 0.001). Several amino acids correlate positively with each other in leaves, nectaries and nectar (*r* = 0.30–0.97; *p* < 0.001), whereby the highest correlations were found in nectar (*r* = 0.70–0.97; *p* < 0.001). Furthermore, the concentration of several amino acids in nectaries correlate with those in nectar (*r* = 0.30–0.65; *p* < 0.001), and the nitrate concentration in leaves positively correlates with the concentration of several amino acids in nectaries (*r* = 0.38–0.72; *p* < 0.001) and in nectar (*r* = 0.33–0.93; *p* < 0.001).

In CAM species, several amino acids correlate positively with each other in leaves (*r* = 0.49–0.88; *p* < 0.001), nectaries (*r* = 0.50–0.89; *p* < 0.001), and nectar (*r* = 0.50–0.88; *p* < 0.001), however, no significant correlations were found between the amino acid concentration in nectaries and nectar (Figure 6B). The correlation matrix of C3 species (Figure 6C) presents a similar pattern as the corresponding matrix for all bromeliad species (Figure 6A). The correlation matrix of trochilophilous C3 plants (Figure 6C) presents almost no interactions in leaves (*r* = 0.55–0.78; *p* < 0.001), whereas correlations can be found in nectaries (*r* = 0.57–0.93; *p* < 0.001) and in nectar (*r* = 0.56–0.99; *p* < 0.001). Further, several amino acids in nectaries correlate with amino acids in nectar (*r* = 0.55-0.93; *p* < 0.001), and the nitrate concentration in leaves positively correlates with the concentration of several amino acids in nectaries (*r* = 0.59-0.85; *p* < 0.001) and in nectar (*r* = 0.67–0.93; *p* < 0.001). In comparison, the matrix of chiropterophilic C3 species (Figure 6D) shows only a few significant correlations of amino acids with each other in leaves and nectar (*r* = 0.55–0.97; *p* < 0.001).

## Discussion

For different plant groups, including bromeliads, it was shown that nectar composition is adapted to pollinator preferences (Baker and Baker, 1983; Petanidou et al., 2006; Abrahamczyk et al., 2017; Tiedge and Lohaus, 2017; Göttlinger et al., 2019; Roguz et al., 2019). A few studies investigated the influence of other physiological properties of plants, such as the photosynthesis type, on nectar composition (Nicolson, 2022) and these studies have mainly focused on dicots whereas, the inclusion of monocots has been neglected. Moreover, as nectar is produced in nectaries, it is highly advisable to investigate the influence of such factors on nectar and nectaries in parallel. Since the nectaries are in turn supplied by the phloem, it is equally required to investigate metabolite concentrations throughout the plant, representatively in the leaves.

### Nectar sugars in relation to the metabolism in nectaries

Sugars, mainly glucose, fructose, and sucrose are the dominant compounds in nectar as well as in nectaries of the analyzed bromeliads (Figure 1A). However, the sugar concentration was generally higher in nectar than in nectaries (Figure 1A). According to the model of Vassilyev (2010), nectar flows by a pressure-driven mass flow in the apoplast of the nectaries, whereas pre-nectar flows from the phloem endings to the secretory cells *via* the symplastic route. Unfortunately, there are no data on the solute concentrations in the apoplastic and symplastic fluid of nectaries as of yet, so the viability of this model cannot be proven. Sugars are actively transported into the apoplast along the path and from the secretory cells, possibly *via* monosaccharide transporters or H^+^ sucrose antiporters (Sherson et al., 2003; Vassilyev, 2010), which leads to high sugar concentrations in the nectar. In the nectaries of *Ananas ananassoides*, abundant mitochondria were found, primarily in the sub-epithelial layers of parenchyma cells (Stahl et al., 2012). This result is in line with the elevated energetic demands due to the secretory processes (Stahl et al., 2012).

The metabolite concentration in the nectary tissue as a total can differ from the concentrations in subdomains of the nectaries or in epithelial cells which are involved in nectar secretion (Lin et al., 2014). Therefore, it cannot be excluded that the concentration of sugars in the nectar secreting tissue and in nectar are similar or even higher in the secreting cells. The determination of the sucrose concentration in nectaries of *Cucurbita pepo* also revealed that the concentration was nearly similar to the sucrose concentration in nectar at the time of maximum nectar secretion (Solhaug et al., 2019). Under these conditions, the transport of sugars into the nectar can be accomplished by facilitated diffusion transporters, such as SWEET9 (Lin et al., 2014; Guo et al., 2018). The fact that *Arabidopsis* mutants lacking SWEET9 do not produce nectar (Lin et al., 2014) underlines the importance of SWEET9 for nectar secretion.

In conclusion, it has not yet been finally clarified what kind of transporters are involved in the secretion of sucrose from the nectary cells into the nectar of bromeliads and further experiments are required to answer this question.

Nectaries are supplied with sucrose *via* the phloem (Fahn, 1979; Lohaus and Schwerdtfeger, 2014) and, depending on the plant species, a specific portion of the delivered sucrose is split into glucose and fructose by sucrose-cleaving enzymes (Tiedge and Lohaus, 2018). In some species, a portion of the produced glucose is transiently stored as starch in nectaries, which is decomposed during nectar secretion (Pacini et al., 2003; Peng et al., 2004; Ren et al., 2007; Tiedge and Lohaus, 2018; Solhaug et al., 2019). This was also shown for *Ananas ananassoides*, whereby the nectary and vascular parenchyma cells of the septal nectaries contained large starch reserves, which can be utilized in the secretory phase (Stahl et al., 2012). However, phloem sugars are also used directly for nectar sugar production (Ning et al., 2017; Solhaug et al., 2019). In the analyzed bromeliads, no correlation between the starch content in nectaries (0.0–17.0 mg starch g^–1^ FW; data not shown) and the sugar concentration in nectar were found (Figure 6). One reason for this could be that starch in nectaries is nearly completely hydrolyzed some hours after anthesis (Stahl et al., 2012; Solhaug et al., 2019).

Moreover, no correlation was found between the starch content in leaves and nectaries, which means that the starch metabolism appears to work independently in both tissues (Figure 6A; Tiedge and Lohaus, 2018). However, the positive correlation between the starch content in leaves and the sugar concentration in nectaries (Figure 6A) suggests that a high carbohydrate level in plants (leaves) is also reflected in nectaries.

The nectar of *Arabidopsis* and other members of the Brassicaceae is hexose-rich (Davis et al., 1998; Lohaus and Schwerdtfeger, 2014). The extracellular hydrolysis of sucrose by nectary-specific cell wall invertases is therefore necessary for the production of nectar (Ruhlmann et al., 2010; Minami et al., 2021). In contrast, the nectar of most bromeliads is sucrose rich and the sucrose-to-hexoses ratio in the nectar is always higher than in the nectaries (Figures 1D–F). Therefore, invertase activity seems to play a less important role in nectar production in bromeliads or other plant species with sucrose rich nectar (Tiedge and Lohaus, 2018; Solhaug et al., 2019).

### Nectar amino acids in relation to the metabolism in nectaries

In all bromeliads, the concentration of amino acids was much lower in nectar than in nectaries (Figure 2). Moreover, the amino acid concentration in nectar is similar to the amino acid concentration in other extracellular fluids in plants, such as the apoplastic fluid in leaves (1–10 mM; Lohaus et al., 2000, 2001). One explanation for the low concentration could be that there is a selective retention of amino acids during nectar secretion; a smaller part of the amino acids, however, leaks from the nectary cells. Another explanation for the origin of amino acids in nectar was described for *Helleborus* (Pacini et al., 2003). Here, the epidermal cells of the nectary die during nectar production and the nectar is enriched with proteins and amino acids from these degenerated cells.

The amino acids in nectaries can be either produced within the nectaries themselves or directly imported from the phloem (Fahn, 1979; Tegeder and Masclaux-Daubresse, 2018). The phloem sap of different plant species contains about 800–1,000 mM sucrose, only traces of hexoses, and about 50–300 mM amino acids (Lohaus, 2022). Compounds in the phloem are transported by mass flow (Patrick, 2013), and as phloem-derived sugars make up a substantial proportion of the sugar content in nectaries (Solhaug et al., 2019), also amino acids may be phloem-derived. The sucrose-to-amino-acid-ratio in the phloem sap is between 3 and 20 (Lohaus, 2022), similar to the corresponding sugar-to-amino-acid-ratio in the nectaries (Table 1). Therefore, it can be assumed that no additional amino acid biosynthesis is necessary in nectaries. However, in floral nectaries of cotton, several genes associated with amino acid biosynthesis were expressed, which may indicate that nectaries of cotton have the potential capacity for the *de novo* biosynthesis of different amino acids (Chatt et al., 2021). In the nectaries of male flowers of *Cucurbita pepo*, also an increased expression and activity of several enzymes involved in the biosynthesis of amino acids at the beginning of nectar secretion was detected (Solhaug et al., 2021). However, the floral nectar of cotton is exceptional with respect to the amino acid composition, as one amino acid—aspartate—is dominant with a proportion of about 90% (Chatt et al., 2021). As for the analyzed bromeliads, the amino acid composition in the nectar is more balanced and reflects the amino acid composition of the nectaries (Figures 3B,C). Therefore, the amount of most amino acids in nectar seems to be already present in the nectaries (Figures 3B,C).

### Nectar inorganic ions in relation to the metabolism in nectaries

In nectaries as well as in nectar, chloride was the dominant anion and potassium the dominant cation (Figure 5). A similar composition of the inorganic ions was found in nectar of other plant species, such as different species of *Nicotiana* (Tiedge and Lohaus, 2017). Potassium and chloride are also the most abundant inorganic ions in the phloem sap of different plant species (Lohaus et al., 2000; Babst et al., 2022; Lohaus, 2022), which suggests that the nectaries are supplied with inorganic ions *via* the phloem. Whereas, the inorganic ion composition is comparable in nectar and nectaries, their concentration is much lower in nectar than in nectaries (Figure 4). Similar to the amino acids, the inorganic ion concentration in nectar (1–30 mM, Figure 4) is comparable to levels found in other extracellular fluids in plants, such as the apoplastic fluid in leaves (15–40 mM inorganic ions; Lohaus et al., 2000, 2001). This indicates that either the export of inorganic ions from the nectaries into the nectar is highly restricted (Göttlinger and Lohaus, 2020), and/or that a smaller proportion of inorganic ions is leaked from the nectaries into the nectar. Moreover, it can be assumed that the transport of inorganic ions in nectaries is apoplastic.

### Correlation between nitrate and amino acids

In most land plants, nitrate is absorbed from the soil through root-localized nitrate transporters (Krapp, 2015). The family of Bromeliaceae contains about 60% epiphytes (Zotz, 2013), for which the main function of their roots is to be anchored upon other plants; water or nutrient uptake occurs *via* leaf-absorbing trichomes. However, in epiphytic bromeliads, both leaves and roots can take up nitrate, with the uptake by the roots being more efficient (Leroy et al., 2019; Gomes et al., 2021). In the analyzed bromeliads, the nitrate concentration in leaves, nectaries, and nectar was generally low (Figure 5A), independent of the potential terrestrial or epiphytic life form (data not shown).

Nevertheless, a positive correlation between the nitrate concentration in leaves and the amino acid concentration in nectaries as well as in nectar was determined (Figure 6A). Absorbed nitrate is transported from the roots to the leaves *via* the xylem, or, in the case of epiphytic bromeliads, it is absorbed by the leaves, where it is metabolized to amino acids (Tegeder and Masclaux-Daubresse, 2018; Leroy et al., 2019). From there, the amino acids can be transported *via* the phloem to the sink tissue, e.g., flowers or nectaries (Tegeder and Hammes, 2018), from where they can be secreted into the nectar. Therefore, it is likely that the more nitrate is taken up, the more amino acids are synthesized and transported to the nectaries and nectar (Figure 6A). Similar results were shown for soil fertilization, whereby fertilized plants showed significantly higher amino acid concentrations in nectar than non-fertilized plants, particularly for glycine and glutamine (Gardener and Gillman, 2001; Gijbels et al., 2015).

In addition, part of the nitrate could also be reduced in nectaries themselves by the activity of nitrate reductase, as it was shown for *Cucurbita pepo* (Solhaug et al., 2021). This part is probably rather small, because phloem sap contains only traces of nitrate and, therefore, the amount of phloem-derived nitrate in the nectaries is probably quite low (Lohaus, 2022). However, the nitrogen metabolism in nectary tissue is not yet understood in its entirety, and the metabolism may vary depending on the plant species and/or ecological factors.

### Metabolite and ion composition in nectar and nectaries is influenced by the photosynthesis type

As nectar compounds are derived directly or indirectly from photosynthesis, different photosynthetic activities of the plant may influence the nectar composition (Pacini et al., 2003). The results of the PERMANOVA showed that the variable photosynthesis (CAM or C3) can explain more data variation of sugars, amino acids, and inorganic ions in nectar than in nectaries or leaves (Table 3). Significantly higher sugar concentrations were found in nectar of C3 species compared to that in CAM species, whereas the sugar concentration in the nectaries or leaves of the corresponding bromeliads were similar (Figure 1B). These results are surprising because the CO_2_-assimilation rate per gram photosynthetic active tissue is generally higher in C3 plants than in CAM plants (Göttlinger et al., 2019), and therefore higher concentrations of sugars in leaves and nectaries of C3 bromeliads would be expected. It is well possible that the similar growing conditions for CAM and C3 plants in the greenhouse caused this effect; in the natural habitats, bromeliads with CAM photosynthesis are to be found in dryer areas and those with C3 photosynthesis in more humid areas (Kessler and Krömer, 2000). Moreover, C3 plants could use the photoassimilates for biomass production rather than for carbon storage.

The investment of plants in the production of nectar can be high and up to 37% of the energy produced by photosynthesis can be used for nectar production (Southwick, 1984). To reduce energy losses caused by nectar secretion, nectar is reabsorbed in some plants at the end of flowering (Stpiczyñska and Nepi, 2006). This is particularly important for plant species with lower CO_2_-assimilation rates or plants that produce large amounts of nectar.

Also, the amino acid concentration in nectar of C3 bromeliads was much higher than in nectar of CAM plants (Figure 2B). In trochilophilous C3 plants, significant correlations between the concentrations of the amino acids in nectar and nectaries as well as significant correlations between the nitrate concentration in leaves and the amino acid concentration in nectaries or nectar were found (Figure 6C). This corresponds to the observation that the amino acid concentration in nectar is influenced by the nitrate content in plants (Gardener and Gillman, 2001; Gijbels et al., 2015). In CAM plants, the corresponding correlations do not exist (Figure 6B). The reason for that may be the generally low nitrate concentration (Supplementary Figure 3) and a higher nitrogen-use efficiency of CAM plants (Winter et al., 1996; Pereira and Cushman, 2019).

### Metabolite and ion composition in nectar and nectaries is influenced by the pollinator type

Nectar of Bromeliaceae is one of the most important energy sources for several hummingbirds and bats in Neotropical regions (Bernadello et al., 1991; Krömer et al., 2006; Göttlinger et al., 2019). The analysis of all data (sugars, amino acids, inorganic ions) by PERMANOVA shows that the pollination type (trochilophilous/chiropterophilous) has an influence on the nectar composition (35%; Table 3), which has already been shown for a large set of bromeliads as well as for other plant species (Baker and Baker, 1983; Tiedge and Lohaus, 2017; Göttlinger et al., 2019). One example for that is the sucrose-rich nectar in hummingbird-pollinated species and the hexose-rich nectar in bat-pollinated species (Figure 1F), which corresponds to the preferences of hummingbirds and bats, respectively (Baker and Baker, 1983). Moreover, the analyses also reveal an influence of the pollination type on the composition of nectaries, albeit to a lesser degree (22%; Table 3); the composition in leaves is not influenced by the pollination type (Table 3). From these results it is obvious that some differences in nectar composition between trochilophilous and chiropterophilous bromeliads are already manifest in the nectaries of these plant groups.

Chiropterophilous C3 species showed a significantly lower sugar concentration and a lower sucrose-to-hexoses-ratio in nectar compared to trochilophilous C3 species (Figures 1C,F; Göttlinger et al., 2019). The same differences can already be seen in nectaries (Figures 1C,F). Therefore, it can be assumed that a large portion of the hexoses in nectar of chiropterophilous bromeliads is already produced in nectaries. Chiropterophilous plant species are basically night-flowering species, and a comparingly higher sucrose cleavage activity within the nectaries was found for night-flowering *Nicotiana* species as well (Tiedge and Lohaus, 2018).

In general, bats and birds do not feed exclusively on nectar, they can also utilize other nitrogen sources (Baker and Baker, 1986). This might be the reason for the amino acid concentration in nectar of vertebrate pollinated species being lower than in insect pollinated species (Tiedge and Lohaus, 2017). As trochilophilous, and even more chiropterophilous species produce large volumes of nectar, it is particularly important to restrain the content of amino acids in the nectar to avoid high nitrogen losses. This corresponds to the lower concentration of amino acids in chiropterophilous bromeliads compared to trochilophilous bromeliads (Figure 2C). The difference in the nectar amino acid concentration is not reflected in the amino acid concentration in nectaries (Figure 2C), which means that the secretion of amino acids into the nectar is more restricted in chiropterophilous bromeliads compared to trochilophilous bromeliads.

The composition of amino acids in nectar for both trochilophilous and chiropterophilous bromeliads is similar to the composition in the nectaries (Supplementary Figures 2B,C). In chiropterophilous bromeliads, only few amino acids have a higher proportion in nectar than in nectaries (Supplementary Figures 2B,C); proline, for example, shows an increased secretion compared to the other amino acids. Despite the fact that proline has been described as an important amino acid in nectar of bee pollinated species (Carter et al., 2006), only small amounts of it were found in the nectar of several bee-pollinated species of *Fritillaria* (Roguz et al., 2019). So far, there is no hint that bats prefer special amino acids (Rodríguez-Peña et al., 2013). Therefore, the amount of proline in nectar seems to be dependent on further factors besides the pollination type.

The concentration of inorganic ions affects the electrolyte balance of some pollinators like nectar feeding birds (Hiebert and Calder, 1983). However, the function of inorganic ions in nectar in relation to the physiology of the pollinators are still poorly investigated (Nicolson, 2022). Inorganic ions are more dominant in nectar of chiropterophilous bromeliads than in nectar of trochilophilous bromeliads (Figure 4C). Similar results were shown for a large collection of further bromeliads and also for other night flowering species (Tiedge and Lohaus, 2017; Göttlinger et al., 2019). The difference in nectar ion concentration is not reflected in nectaries or leaves (Figure 4C). It is possible that the relationship between the xylem and phloem transport plays a decisive role for this matter. Furthermore, the physiological or biochemical processes that lead to higher concentrations of inorganic ions in nectar of bat-pollinated or other night-flowering plants require further study.

## Conclusion

In summary, it was possible to gain new insights into the origins of nectar compounds and in the secretion processes of metabolites and inorganic ions from the nectaries into nectar for several bromeliads with different pollination types, photosynthesis types, or life forms. The differences in the composition and concentration of sugars in nectaries and nectar are probably due to metabolism of sugars during secretion and different transport processes. The transport of amino acids or inorganic ions from nectaries to nectar is more restricted. Yet, the more nitrate is taken up, the more amino acids are produced in leaves and transported into nectaries and nectar. The photosynthesis type (CAM/C3) and the pollination type (trochilophilous/chiropterophilous) can explain more data variation in nectar than in nectaries and leaves, but the pollinator type has a stronger influence on the nectar or nectary composition than the photosynthesis type. However, more research is needed to fully understand the origin of nectar compounds, especially with respect to monocots, CAM plants, and plant species with other physiological properties or life forms.

## Data availability statement

The original contributions presented in this study are included in the article/Supplementary material, further inquiries can be directed to the corresponding author.

## Author contributions

GL planned and designed the research. TG designed and performed the experiments. TG and GL carried out the data analysis and wrote the manuscript. Both authors contributed to the article and approved the submitted version.

## Footnotes

1
www.r-project.org
