# Supplementary material for: Comparative analyses of the metabolite and ion concentrations in nectar, nectaries, and leaves of 36 bromeliads with different photosynthesis and pollinator types
Source: Front Plant Sci. 2022 Aug 26;13:987145. doi: 10.3389/fpls.2022.987145 (PMC9459329; doi:10.3389/fpls.2022.987145)
Supplement: Supplementary file 3 [file Image_2.pdf]

Supplementary Material

Comparative analyses of the metabolite and ion concentrations in nectar, nectaries, and leaves of 36 bromeliads with different photosynthesis and pollinator types

Author: Thomas Göttlinger*, Gertrud Lohaus

***Correspondence:** Thomas Göttlinger (goettlinger@uni-wuppertal.de)

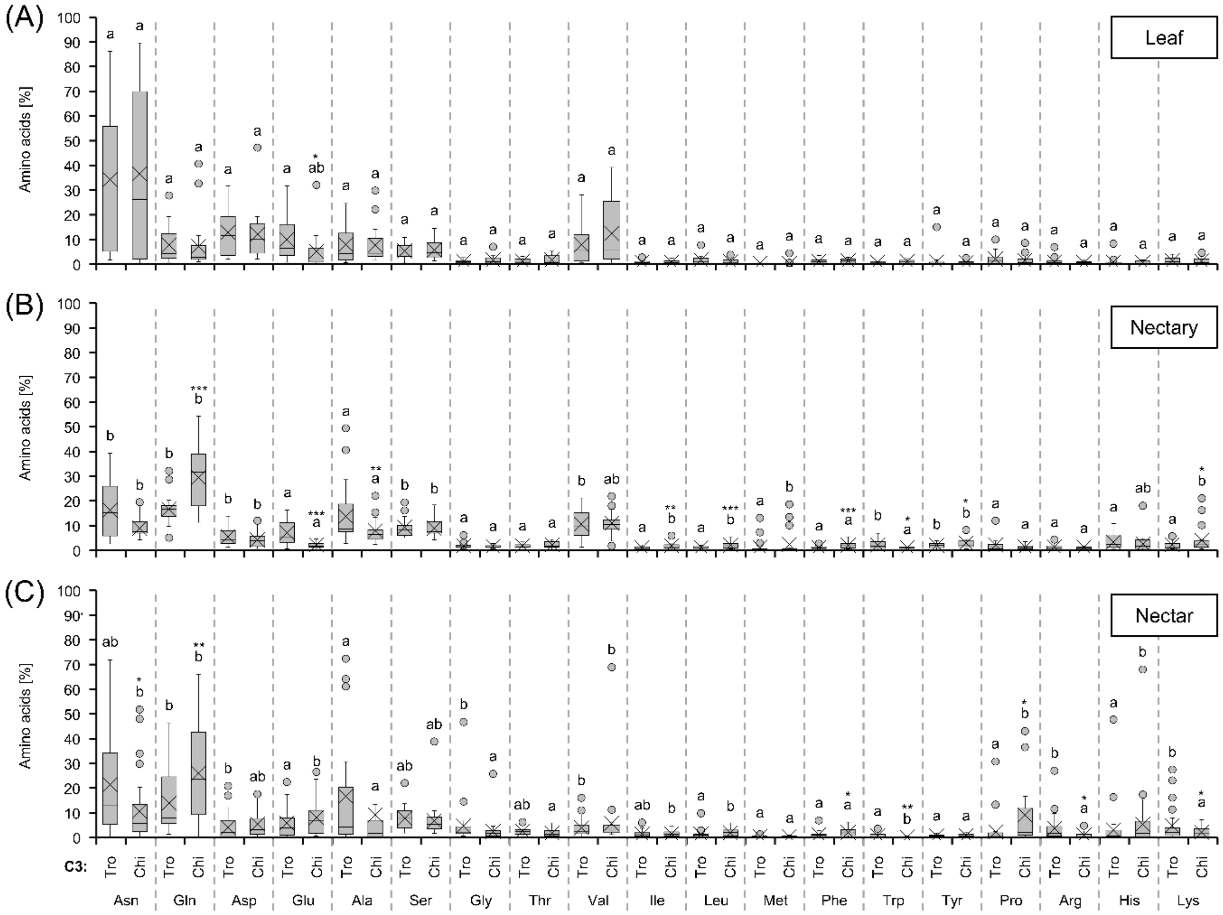

Supplementary Figure S2: Percentage of amino acids in leaf (A), nectary (B) and nectar (C) of C3 plants separated by pollinator type including 11 trochilophilous (tro) and 11 chiropterophilous (chi) species (n = 3). Different letters represent significant differences in sum of amino acids between leaf, nectary and nectar (Tukey's HSD; $p < 0.05$). The asterisks show different levels of significance between the photosynthesis and pollinator types (* $p < 0.05$, ** $p < 0.01$, *** $p < 0.001$).
