# Supplementary material for: Comparative analyses of the metabolite and ion concentrations in nectar, nectaries, and leaves of 36 bromeliads with different photosynthesis and pollinator types
Source: Front Plant Sci. 2022 Aug 26;13:987145. doi: 10.3389/fpls.2022.987145 (PMC9459329; doi:10.3389/fpls.2022.987145)
Supplement: Supplementary file 6 [file Image_5.pdf]

## *Supplementary Material*

# Comparative analyses of the metabolite and ion concentrations in nectar, nectaries, and leaves of 36 bromeliads with different photosynthesis and pollinator types

**Author: Thomas Göttlinger*, Gertrud Lohaus**

***Correspondence:** Thomas Göttlinger (goettlinger@uni-wuppertal.de)

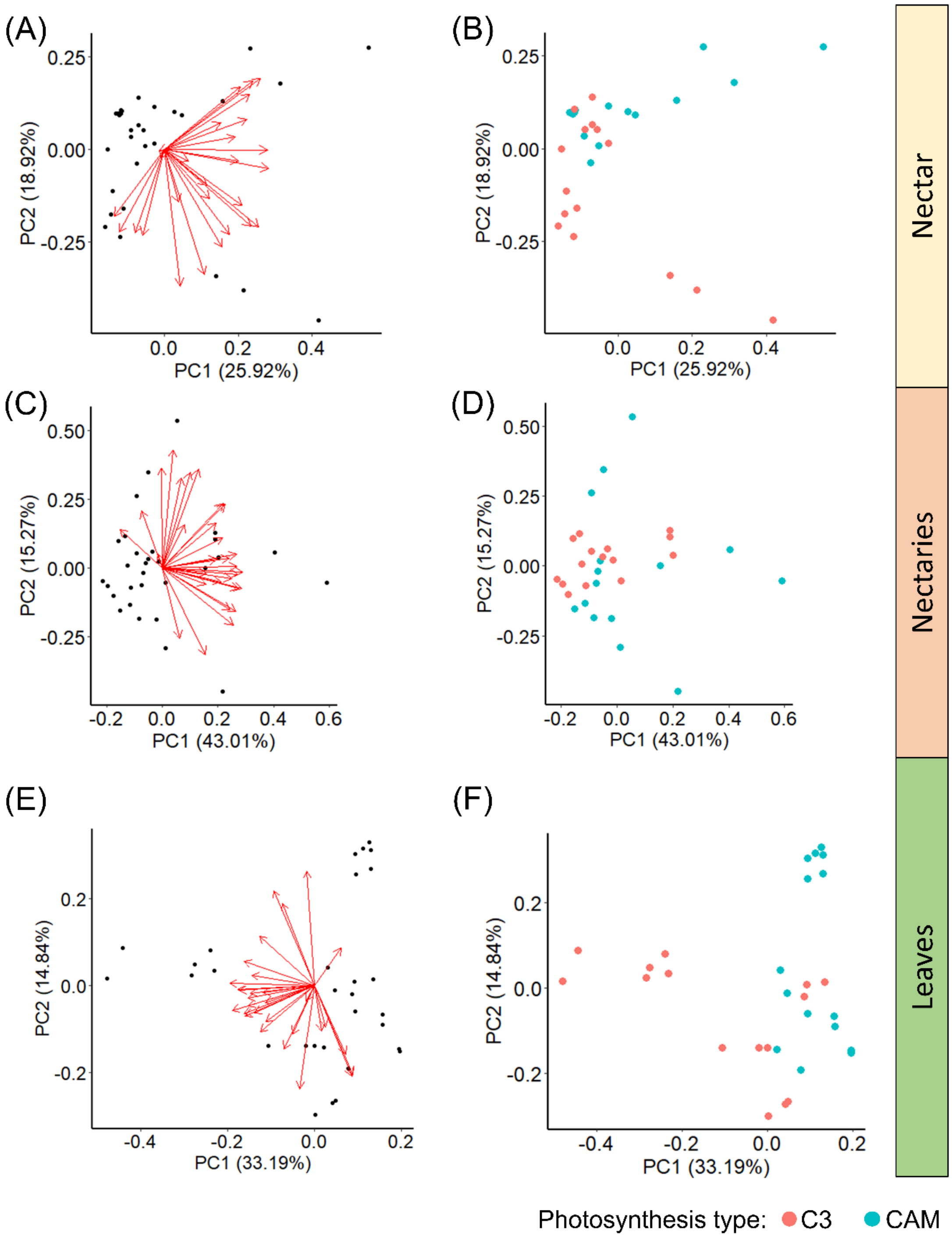

**Supplementary Figure S5:** Loadings and scatterplot of Principal Component Analysis (PCA) in rotated space (Photosynthesis type). In each case, five species from different genera were selected based on the photosynthesis type (C3 or CAM). CAM: *Aechmea fasciata*, *Billbergia morelli*, *Portea petropolitana*, *Quesnelia quesneliana*, *Tillandsia flabellata*; C3: *Alcantarea regina*, *Guzmania melinonis*, *Pitcairnia olivia-estevae*, *Tillandsia malzinei*, *Vriesea guttata*. All species belongs to trochilophilous pollinator type. (A, C, E) The loading plot illustrates the variables loaded as vectors in PCA space. Thereby, the principal components (PC1 & PC2) describe the dataset variation. (B, D, F) In the scatterplot of PCA presents the data grouped by photosynthesis type (colors). The plots represent the data of nectar (A & B), nectaries (C & D) and leaf (E & F).
