# Supplementary material for: Comparative analyses of the metabolite and ion concentrations in nectar, nectaries, and leaves of 36 bromeliads with different photosynthesis and pollinator types
Source: Front Plant Sci. 2022 Aug 26;13:987145. doi: 10.3389/fpls.2022.987145 (PMC9459329; doi:10.3389/fpls.2022.987145)
Supplement: Supplementary file 7 [file Image_6.pdf]

Supplementary Material

Comparative analyses of the metabolite and ion concentrations in nectar, nectaries, and leaves of 36 bromeliads with different photosynthesis and pollinator types

Author: Thomas Göttlinger*, Gertrud Lohaus

***Correspondence:** Thomas Göttlinger (goettlinger@uni-wuppertal.de)

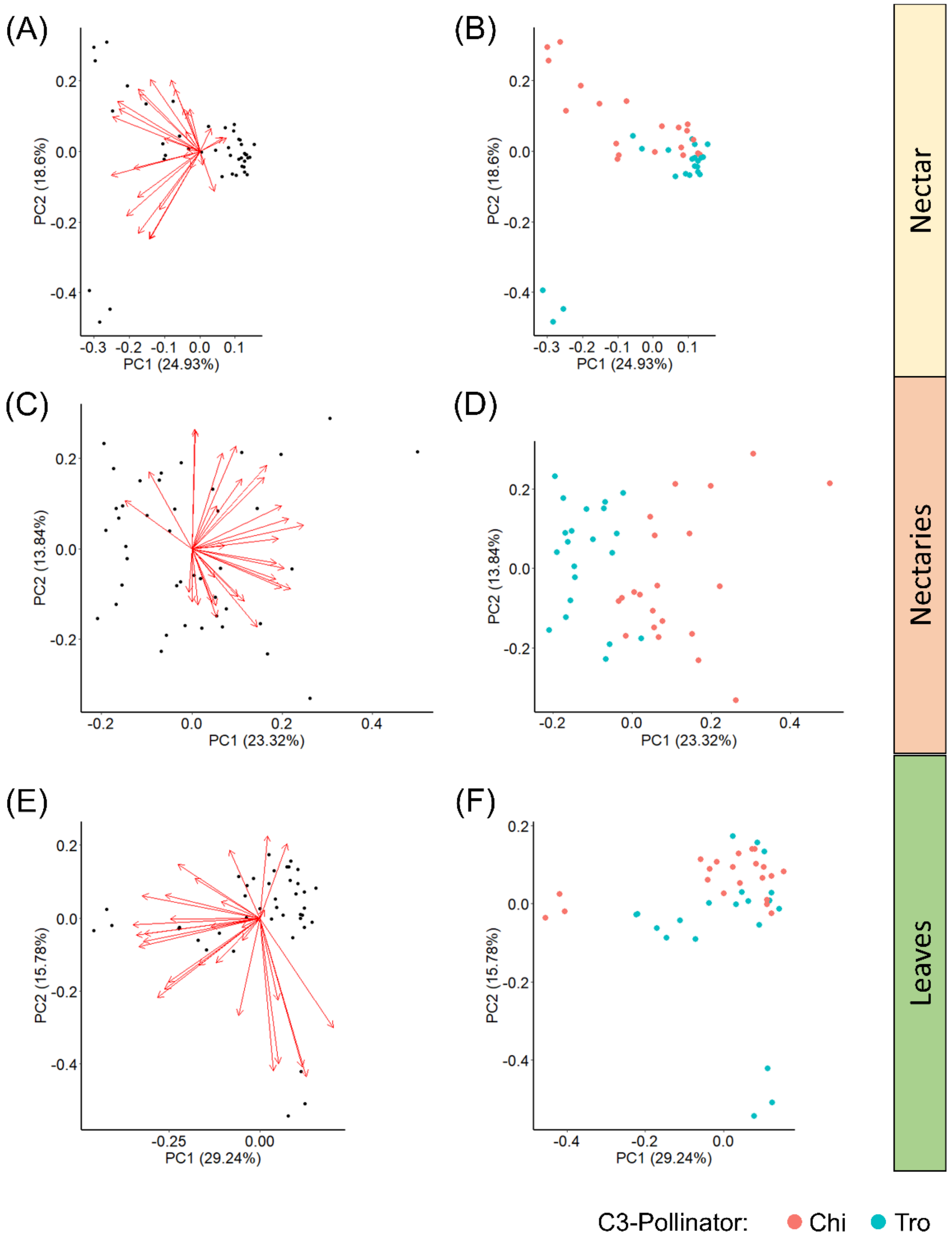

Supplementary Figure S6: Loadings and scatterplot of Principal Component Analysis (PCA) in rotated space (Pollinator). In each case, seven C3-species from five different genera were selected based on the pollinator (Chi = chiropterophilous or Tro = trochilophilous). Chi: *Alcantarea imperalis*, *Guzmania calothyrsus*, *Pitcairnia wendlandii*, *Pseudalcantarea viridiflora*, *Tillandsia rauhii*, *Vriesea unlilateralis*, *Werauhia werckleana*; Tro: *Alcantarea regina*, *Billbergia euphemiae*, *Guzmania melinonis*, *Lutheria splendens*, *Pitcairnia corallina*, *Tillandsia malzinei*, *Vriesea guttata*. (A, C, E) The loading plot illustrates the variables loaded as vectors in PCA space. Thereby, the principal components (PC1 & PC2) describe the dataset variation. (B, D, F) In the scatterplot of PCA presents the data grouped by pollinator (colors). The plots represent the data of nectar (A & B), nectaries (C & D) and leaf (E & F).
