# Supplementary material for: Comparative analyses of the metabolite and ion concentrations in nectar, nectaries, and leaves of 36 bromeliads with different photosynthesis and pollinator types
Source: Front Plant Sci. 2022 Aug 26;13:987145. doi: 10.3389/fpls.2022.987145 (PMC9459329; doi:10.3389/fpls.2022.987145)
Supplement: Supplementary file 8 [file Table_1.pdf]

Supplementary Material

Comparative analyses of the metabolite and ion concentrations in nectar, nectaries, and leaves of 36 bromeliads with different photosynthesis and pollinator types

Author: Thomas Göttlinger*, Gertrud Lohaus

***Correspondence:** Thomas Göttlinger (goettlinger@uni-wuppertal.de)

Supplementary Table 1: Overview of 36 examined Bromeliaceae species.

Species	Subfamily	Pollination type	Growing type	CAM or C3 [1]
Aechmea fasciata (Lindl.) Baker	Bromelioideae	Tro [2]	E	CAM
Aechmea gamosepala Wittm.	Bromelioideae	Tro [3]	E	CAM
Aechmea miniata discolor (Beer) Beer ex Baker	Bromelioideae	Tro [4]	E	CAM
Aechmea nudicaulis (L.) Griseb.	Bromelioideae	Tro [4]	E	CAM
Aechmea orlandiana L.B.Sm.	Bromelioideae	Tro [4]	T	CAM
Aechmea recurvata (Klotzsch) L.B.Sm.	Bromelioideae	Tro [3]	T	CAM
Alcantarea imperialis (Carriere) Harms	Tillandsioideae	Chi [5]	T	C3
Alcantarea regina (Vell.) Harms	Tillandsioideae	Tro [6]	T	C3
Billbergia euphemiae E.Morren	Bromelioideae	Tro [7]	E	C3
Billbergia fosteriana L.B.Sm.	Bromelioideae	Tro [6]	E	CAM
Billbergia morelii Brongn.	Bromelioideae	Tro [8]	E	CAM
Billbergia vittata Brongn. ex C.Morel	Bromelioideae	Tro [9]	E	CAM
Guzmania calothyrsus Mez	Tillandsioideae	Chi [10]	E	C3
Guzmania cylindrica L.B.Sm.	Tillandsioideae	Chi [6]	E	C3
Guzmania killipiana L.B.Sm.	Tillandsioideae	Chi [4]	E	C3
Guzmania melinonis Regel	Tillandsioideae	Tro [10]	E	C3
Lutheria splendens (Brongn.)	Tillandsioideae	Tro [4]	E	C3
Pitcairnia corallina Linden & André	Pitcairnioideae	Tro [7]	T	C3
Pitcairnia longissimiflora Ibisch, R.Vasquez & E.Gross	Pitcairnioideae	Chi [6]	T	C3
Pitcairnia maidifolia (C.Morren) Decne. ex Planch.	Pitcairnioideae	Tro [6]	T	C3
Pitcairnia olivia-estevae J.R.Grant	Pitcairnioideae	Tro [6]	T	C3
Pitcairnia recurvata (Scheidw.) K.Koch	Pitcairnioideae	Chi [11]	T	C3
Pitcairnia sceptigera Mez	Pitcairnioideae	Tro [6]	T	C3
Pitcairnia wendlandii Baker	Pitcairnioideae	Chi [6]	T	C3
Portea petropolotana (Wawra) Mez	Bromelioideae	Tro [7]	T	CAM
Pseudalcantarea viridiflora (Beer) Pinzón & Barfuss	Pitcairnioideae	Chi [2]	E	C3
Quesnelia quesneliana (Brongn.) L.B.Sm.	Bromelioideae	Tro [12]	E	CAM
Tillandsia flabellata Baker	Tillandsioideae	Tro [4]	E	CAM
Tillandsia funckiana Baker	Tillandsioideae	Tro [6]	E	CAM
Tillandsia ionantha Planch.	Tillandsioideae	Tro [6]	E	CAM
Tillandsia malzinei (E.Morren) Baker	Tillandsioideae	Tro [6]	E	C3
Tillandsia rauhii L.B.Sm.	Tillandsioideae	Chi [6]	E	C3
Vriesea guttata Linden & Andre	Tillandsioideae	Tro [6]	E	C3
Vriesea maxoniana (L.B.Sm.) L.B.Sm.	Tillandsioideae	Tro [10]	E	C3
Vriesea unilateralis (Baker) Mez	Tillandsioideae	Chi [4]	E	C3
Werauhia werckleana (Mez) J.R.Grant	Tillandsioideae	Chi [6]	E	C3

Pollination type: Chi = chiropterophilous, Tro = trochilophilous. Growing type: T = terrestrial, E = epiphytic.

References

1. Crayn D.M., Winter K., Schulte K., Smith J.A.C. (2015) Photosynthetic pathways in Bromeliaceae. Phylogenetic and ecological significance of CAM and C_3 based on carbon isotope ratios for 1893 species. *Botanical Journal of the Linnean Society*, 178, 169–221. doi: 10.1111/boj.12275
2. Benzing D.H. (2000) Bromeliaceae: profile of an adaptive radiation. *Cambridge University Press*. doi: 10.1017/CBO9780511565175
3. Martín González A.M. et al. (2015) The macroecology of phylogenetically structured hummingbird-plant networks. *Global Ecology and Biogeography*, 24, 1212–1224; doi: 10.1111/geb.12355
4. Krömer T., Kessler M., Lohaus G., Schmidt-Lebuhn A.N. (2008) Nectar sugar composition and concentration in relation to pollination syndromes in Bromeliaceae. *Plant Biology*, 10, 502–511. doi: 10.1111/j.1438-8677.2008.00058.x
5. Santos V.L.D., Versieux L.M., Wanderley M.D.G.L., Da Luz C.F.P. (2017) Pollen morphology of *Alcantarea* giant bromeliads (Bromeliaceae, Tillandsioideae). *Grana*, 57, 117–136. doi: 10.1080/00173134.2017.1310920
6. Göttlinger T., Schwerdtfeger M., Tiedge K., Lohaus G. (2019) What do nectarivorous bats like? Nectar composition in Bromeliaceae with special emphasis on bat-pollinated species. *Frontiers in Plant Science*, 10, 205. doi: 10.3389/fpls.2019.00205
7. Martinelli G. (1995) Reproductive biology of Bromeliaceae in the atlantic rainforest of southeastern Brazil. PhD thesis.
8. Tavares D.C., Freitas L., Gaglianone M.C. (2016) Data compilation of hummingbird-pollinated plant species in the Brazilian Atlantic rain forest. *PANGAEA*. doi: 10.1594/PANGAEA.859056
9. Rodrigues L.C., Rodrigues M. (2014) Flowers visited by hummingbirds in the open habitats of the southeastern brazilian mountaintops. Species composition and seasonality. *Brazilian Journal of Biology*, 74, 659–676. doi: 10.1590/bjb.2014.0097
10. Krömer T., Kessler M., Herzog S.K. (2006) Distribution and flowering ecology of bromeliads along two climatically contrasting elevational transects in the Bolivian Andes. *Biotropica*, 38, 183–195. doi: 10.1111/j.1744-7429.2006.00124.x
11. Aguilar-Rodríguez P.A., Tschapka M., García-Franco J.G., Krömer T. & MacSwiney G M.C. (2019) Bromeliads going batty: pollinator partitioning among sympatric chiropterophilous Bromeliaceae. *AoB PLANTS*, 11, 37. doi: 10.1093/aobpla/plz014
12. Fonseca L.C.N., Vizentin-Bugoni J., Rech A.R., Alves M.A.S. (2015) Plant-hummingbird interactions and temporal nectar availability in a restinga from Brazil. *Anais da Academia Brasileira de Ciencias*, 87, 2163–2175. doi: 10.1590/0001-3765201520140349
